# Supplementary figures and images for: Joint spatiotemporal modelling reveals seasonally dynamic patterns of Japanese encephalitis vector abundance across India
Source: PLoS Negl Trop Dis. 2022 Feb 22;16(2):e0010218. doi: 10.1371/journal.pntd.0010218 (PMC8896663; doi:10.1371/journal.pntd.0010218)

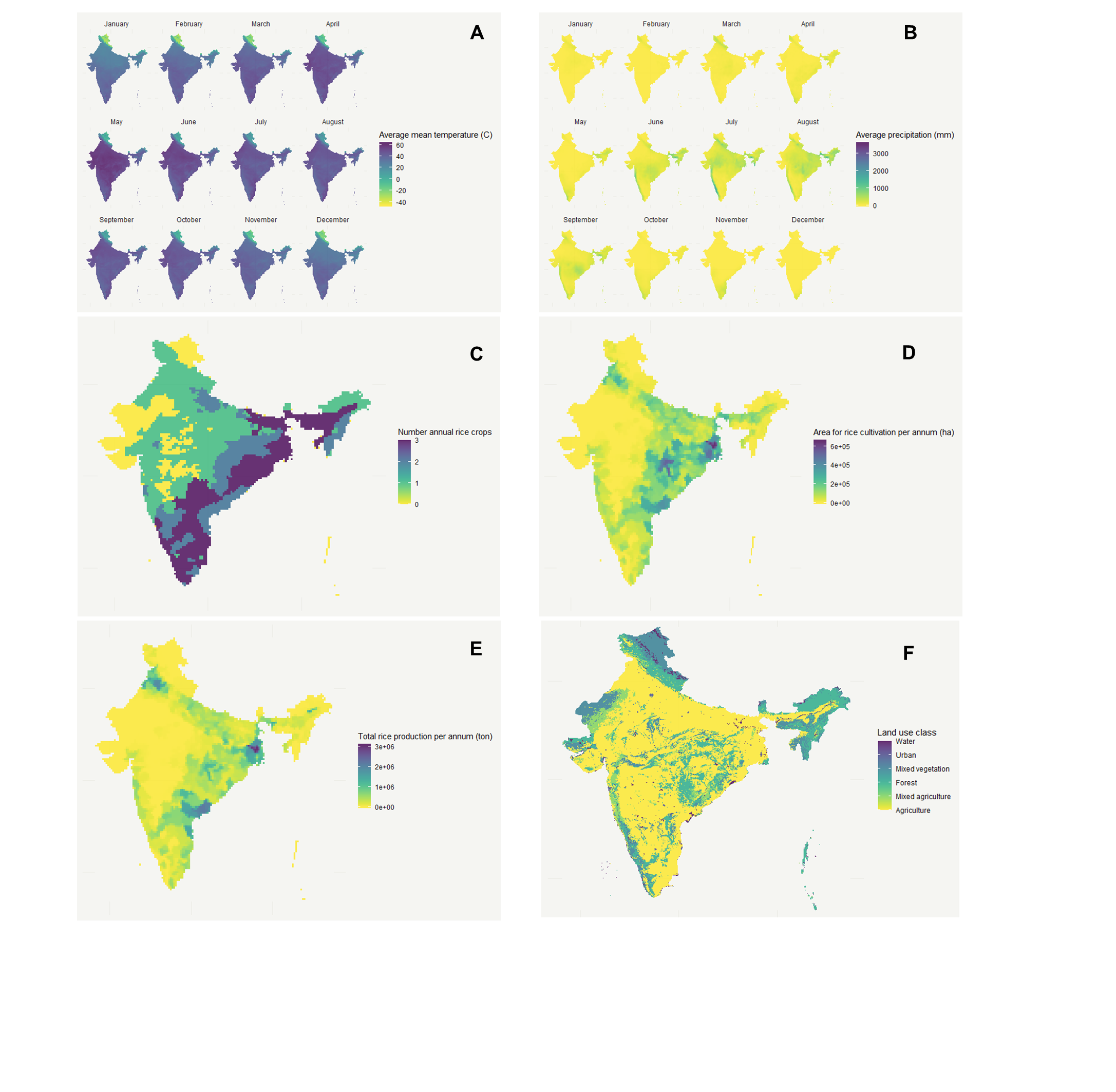

Supplement: S1 Fig — (A) average mean temperature per month (0C) (example given for the year 2005); (B) average precipitation per month (mm) (example given for the year 2005); (C) number of rice crop rotations per year (average for period 2010–12); (D) total annual rice area cultivated per year in hectares (average for period 2010–12); (E) total rice produced per year in tonnes (average for period 2010–12); (F) land use classes (example given for the year 2005). Source of base layer https://gadm.org. (TIF) [file pntd.0010218.s001.tif]

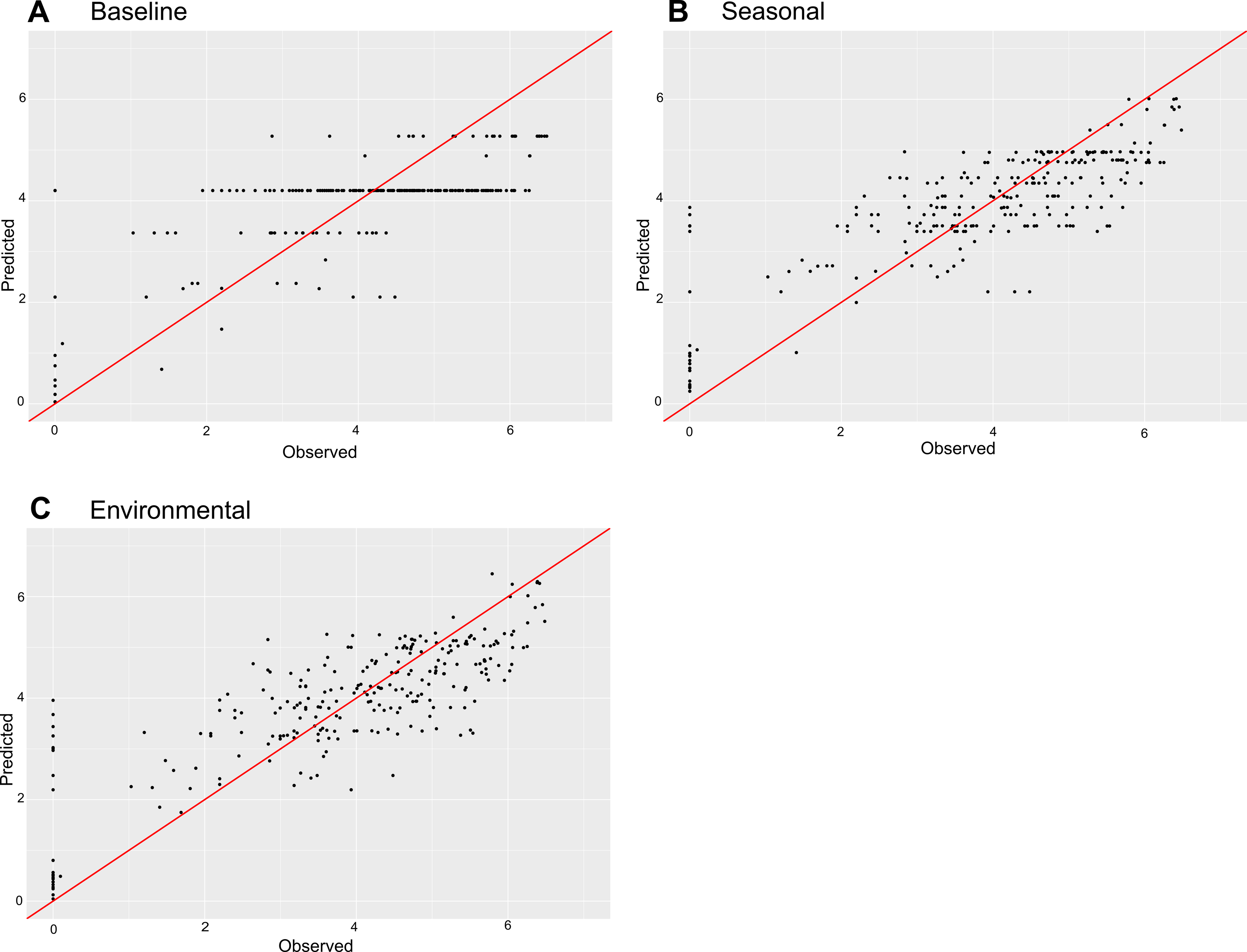

Supplement: S2 Fig — Plots show observed data against model predicted values, and the red line shows the expectation if observed equals predicted for each model: (A) baseline (spatial effects and study- level random effects), (B) seasonal (spatial, seasonal, and random effects), (C) environmental (spatial, seasonal, and random effects and environmental covariates). (TIF) [file pntd.0010218.s002.tif]

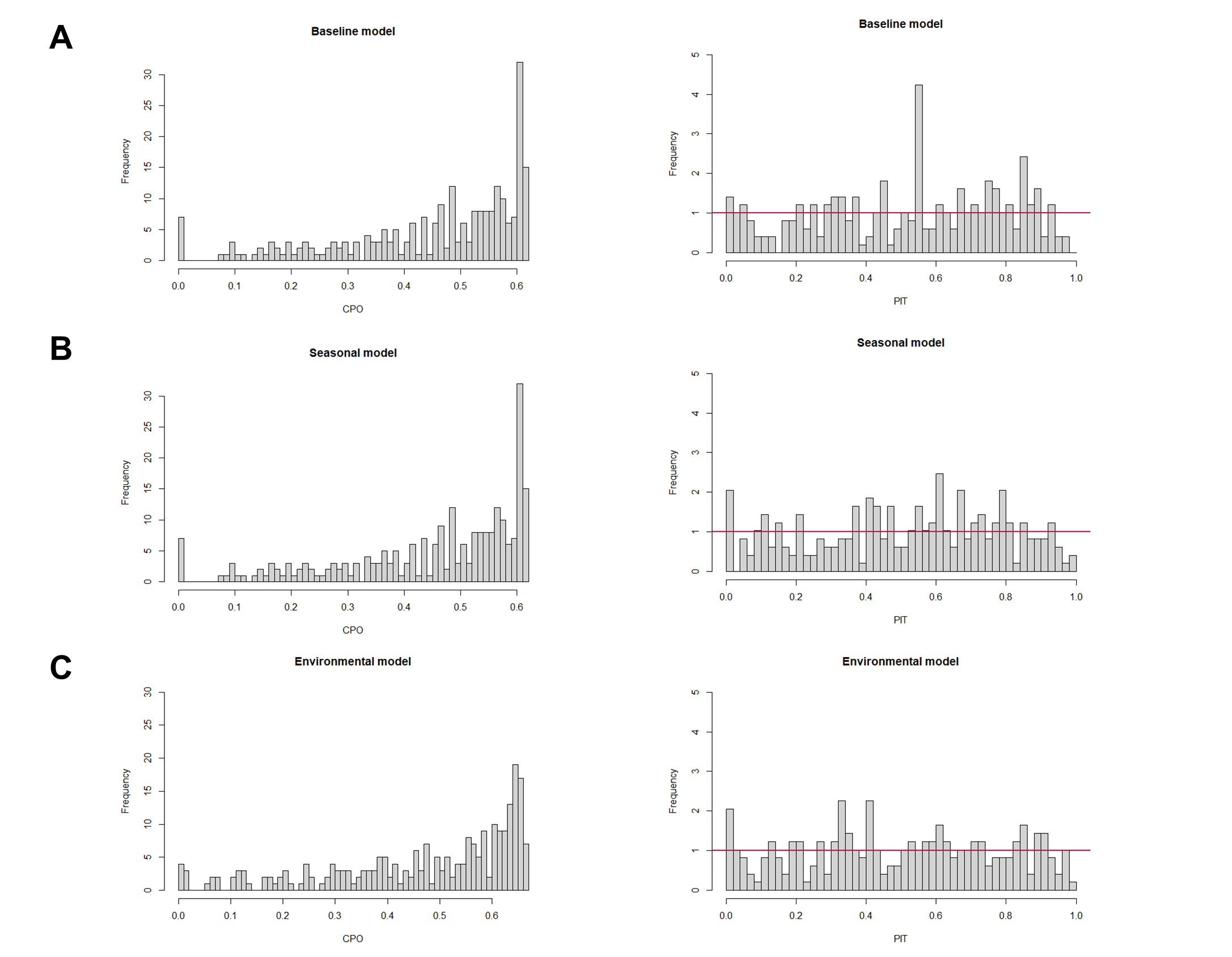

Supplement: S3 Fig — Plots show CPO and PIT histograms, with the red line indicating the level of the of the different values if their distribution was uniform: (A) baseline (spatial effects and study- level random effects), (B) seasonal (spatial, seasonal and random effects), (C) environmental (spatial, seasonal and random effects and environmental covariates). (TIF) [file pntd.0010218.s003.tif]

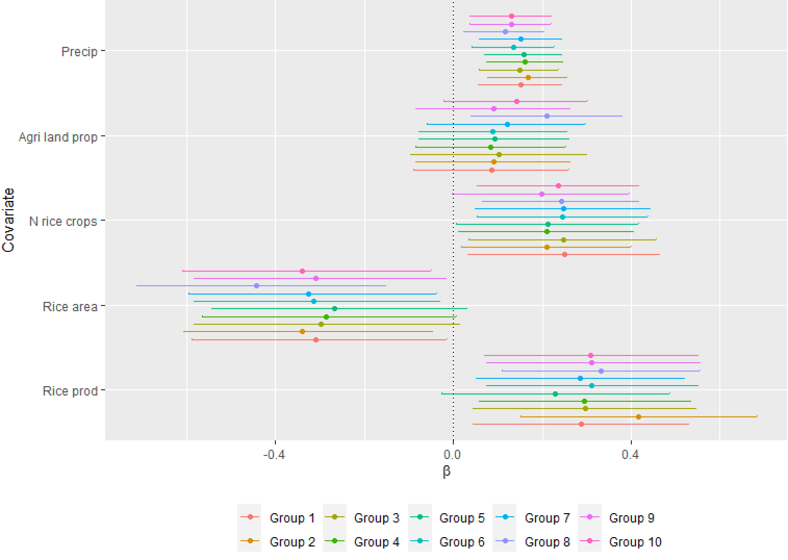

Supplement: S4 Fig — We tested the sensitivity of fixed effects estimates to random (10-fold) subsampling. Points and error bars show posterior marginal parameter distributions for each hold-out model (median and 95% quantile range), with colour denoting hold-out group. Directionality and magnitude of fixed-effects estimates are robust to all tests. (TIF) [file pntd.0010218.s004.tif]

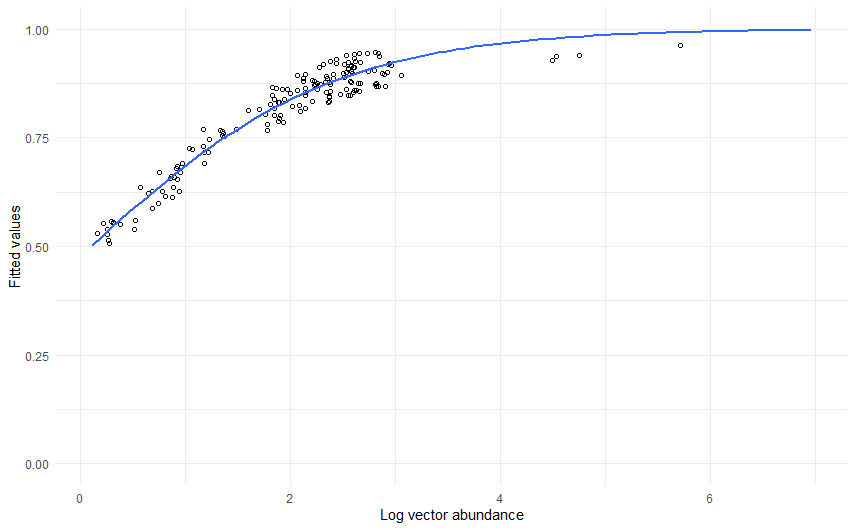

Supplement: S5 Fig — Vertical axis displays model predicted JE outbreak probability, and vertical axis gives predicted vector abundance on the log scale. Smooth line highlights the non-linear relationship of JE outbreak probability to predicted vector abundance with a one-month lag. (TIF) [file pntd.0010218.s005.tif]
