# Supplementary material for: Joint spatiotemporal modelling reveals seasonally dynamic patterns of Japanese encephalitis vector abundance across India
Source: PLoS Negl Trop Dis. 2022 Feb 22;16(2):e0010218. doi: 10.1371/journal.pntd.0010218 (PMC8896663; doi:10.1371/journal.pntd.0010218)
Supplement: S1 Table — The table includes the study from which the data were extracted, the state or union territory in India in which the survey was conducted, the year of the survey, the type of data collected, the survey method, the total number of months that were surveyed, the number of sampling sites per study and the total number of datapoints (occurrence and abundance) generated from the study. (DOCX) [file pntd.0010218.s006.docx]

**S1 Table. Vector surveillance data used in analyses.** The table includes the study from which the data were extracted, the state or union territory in India in which the survey was conducted, the year of the survey, the type of data collected, the survey method, the total number of months that were surveyed, the number of sampling sites per study and the total number of datapoints (occurrence and abundance) generated from the study.

| Study  reference | Surveyed state/ union territory | Date range | Type of vector surveillance data* | Survey method for adult mosquitos | Total number of months surveyed | Number of sampling sites | Number of occurrence datapoints | Number of abundance datapoints | Total number of datapoints |
| --- | --- | --- | --- | --- | --- | --- | --- | --- | --- |
| (1) | Andhra Pradesh | 2003-2004 | OC | Aspirator | 1 | 2 | 3 | - | 3 |
| (2) | Assam | 1993 | OC | Aspirator | 1 | 1 | 1 | - | 1 |
| (3) | Assam | 2011 | OC | Indoor light trap, aspirator | 1 | 3 | 3 | - | 3 |
| (4) | Delhi | 2011 | OC, AB | Aspirator, net sweeping | 1 | 2 | 2 | 2 | 4 |
| (5) | Goa | 2006 | OC, AB | Human landing catch | 1 | 1 | 1 | 1 | 2 |
| (6) | Gujarat | 2003 | OC, AB | Aspirator | 1 | 1 | 1 | 1 | 2 |
| (7) | Gujarat,  Uttar Pradesh | 2005 | OC, AB | Aspirator | 1 | 2 | 2 | 2 | 4 |
| (8) | Karnataka, Kerala | 2003 | OC | Aspirator | 1 | 2 | 2 | - | 2 |
| (9) | Karnataka, Maharashtra, Tamil Nadu | 2007 | OC | Aspirator | 1 | 3 | 3 | - | 3 |
| (10) | Kerala | 2011 | OC, AB | Aspirator | 1 | 1 | 1 | 1 | 2 |
| (11) | Odisha | 1990-1991 | OC, AB | Aspirator | 24 | 1 | 24 | 18 | 42 |
| (12) | Odisha | 1993 | OC, AB | Aspirator | 1 | 1 | 1 | 1 | 2 |
| (13) | Odisha | 2000 | OC, AB | Light trap, aspirator | 1 | 2 | 2 | 1 | 3 |
| (14) | Tamil Nadu | 1998-2000 | OC, AB | Aspirator | 23 | 2 | 46 | 23 | 69 |
| (15) | Tamil Nadu | 2003-2006 | OC, AB | Hand catch | 4 | 9 | 20 | 4 | 24 |
| (16) | Tamil Nadu | 2006-2011 | OC, AB | Aspirator | 60 | 3 | 180 | 180 | 360 |
| (17) | Tamil Nadu | 2007-2008 | OC | Not described | 1 | 1 | 2 | - | 2 |
| (18) | Tamil Nadu | 2011-2012 | OC, AB | Aspirator | 21 | 1 | 21 | 21 | 42 |
| (19) | Telangana | 2003 | OC, AB | Aspirator | 1 | 6 | 6 | 6 | 12 |
| (20) | Uttar Pradesh | 1991 | OC | Aspirator | 1 | 4 | 4 | - | 4 |
| (21) | Uttar Pradesh | 1991-2000 | OC | Aspirator | 1 | 1 | 3 | - | 3 |
| (22) | Uttar Pradesh | 2011 | OC, AB | Aspirator | 1 | 2 | 2 | 2 | 4 |
| (23) | West Bengal | 2000 | OC | Light trap, aspirator | 1 | 2 | 2 | - | 2 |
| (24) | West Bengal | 2011-2012 | OC, AB | Aspirator | 2 | 4 | 8 | 4 | 12 |
| Totals |  |  |  |  |  | **54** | **340** | **267** | **607** |

**OC = occurrence data; AB = abundance data*

**References**

1. Rajavel AR, Natarajan R, Vaidyanathan K. Mosquitoes of the mangrove forests of India: Part 4--Coringa, Andhra Pradesh. J Am Mosq Control Assoc. 2006 Dec;22(4):579–81.

2. Bhattacharyya DR, Handique R, Dutta LP, Dutta P, Doloi P, Goswami BK, et al. Host feeding patterns of Culex vishnui sub group of mosquitoes in Dibrugarh district of Assam. J Commun Dis. 1994;26(3):133–8.

3. Dhiman S, Rabha B, Talukdar PK, Das NG, Yadav K, Baruah I, et al. DDT & deltamethrin resistance status of known Japanese encephalitis vectors in Assam, India. Indian J Med Res. 2013 Dec;138(6):988–94.

4. First indigenous transmission of Japanese Encephalitis in urban areas of National Capital Territory of Delhi, India - Kumari - 2013 - Tropical Medicine &amp; International Health - Wiley Online Library [Internet]. [cited 2021 Feb 9]. Available from: https://onlinelibrary.wiley.com/doi/full/10.1111/tmi.12104

5. Korgaonkar NS, Kumar A, Yadav RS, Kabadi D, Dash AP. Mosquito biting activity on humans & detection of Plasmodium falciparum infection in Anopheles stephensi in Goa, India. Indian J Med Res. 2012;135:120–6.

6. Rajavel a R, Natarajan R. Mosquitoes of the mangrove forests of India: part 7–an overview. J Am Mosq Control Assoc. 2008;24(4):478–88.

7. Srivastava VK, Singh A, Thapar BR. Field evaluation of malathion fogging against Japanese encephalitis vector, Culex tritaeniorhynchus. J Vector Borne Dis. 2008 Sep;45(3):249–50.

8. Rajavel AR, Natarajan R, Vaidyanathan K. Mosquitoes of the mangrove forests of India: Part 6--Kundapur, Karnataka and Kannur, Kerala. J Am Mosq Control Assoc. 2006 Dec;22(4):582–5.

9. Kanojia PC, Paingankar MS, Patil A a, Gokhale MD, Deobagkar DN. Morphometric and allozyme variation in Culex tritaeniorhynchus mosquito populations from India. J Insect Sci Online. 2010;10(138):138.

10. Thenmozhi V, Paramasivan R, Samuel PP, Kamaraj T, Balaji T, Dhananjeyan KJ, et al. Japanese encephalitis virus isolation from mosquitoes during an outbreak in 2011 in Alappuzha district, Kerala. J Vector Borne Dis. 2013 Sep;50(3):229–31.

11. Yadav R, Sharma V, Chand S. Mosquito breeding and resting in treeholes in a forest ecosystem in Orissa. Indian J Malariol. 1997;

12. Dash AP, Chhotray GP, Mahapatra N, Hazra RK. Retrospective analysis of epidemiological investigation of Japanese encephalitis outbreak occurred in Rourkela, Orissa, India. Southeast Asian J Trop Med Public Health. 2001;32(1):137–9.

13. Rajavel AR, Natarajan R, Vaidyanathan K. Mosquitoes of the mangrove forests of India: Part 1--Bhitarkanika, Orissa. J Am Mosq Control Assoc. 2005 Jun;21(2):131–5.

14. Rajendran R, Thenmozhi V, Tewari SC, Balasubramanian A, Ayanar K, Manavalan R, et al. Longitudinal studies in South Indian villages on Japanese encephalitis virus infection in mosquitoes and seroconversion in goats. Trop Med Int Health TM IH. 2003;8(2):174–81.

15. Samuel PP, Arunachalam N, Rajendran R, Leo SVJ, Ayanar K, Balasubramaniam R, et al. Temporal Variation in the Susceptibility of Culex tritaeniorhynchus (Diptera: Culicidae) to Japanese Encephalitis Virus in an Endemic Area of Tamil Nadu, South India. Vector-Borne Zoonotic Dis. 2010 Apr 28;10(10):1003–8.

16. Samuel PP, Ramesh D, Thenmozhi V, Nagaraj J, Muniaraj M, Arunachalam N. Japanese Encephalitis vector abundance and infection frequency in Cuddalore District, Tamil Nadu, India: a five-year longitudinal study. J Entomol Acarol Res. 2016 Dec 19;48(3):366–71.

17. Paramasivan R, Dhananjeyan KJ, Pandian RS. A preliminary report on DNA barcoding and phylogenetic relationships of certain public health important mosquito species recorded in rural areas of south India. J Vector Borne Dis. 2013 Jun;50(2):144–6.

18. Tyagi B, Samuel P, Thenmozhi V, Nagaraj J, Ramesh D, Selvi S, et al. Determination of critical density and vectorial capacity for Culex tritaeniorhynchus Giles, 1901 (Diptera: Culicidae), the primary vector for Japanese encephalitis in southern India. Int J Mosq Res. 2016;3(2):39–46.

19. Das BP, Lal S, Saxena VK. Outdoor resting preference of Culex tritaeniorhynchus, the vector of Japanese encephalitis in Warangal and Karim Nagar districts, Andhra Pradesh. J Vector Borne Dis. 2004 Jun;41(1–2):32–6.

20. Kanojia PC, Shetty PS, Geevarghese G. A long-term study on vector abundance & seasonal prevalence in relation to the occurrence of Japanese encephalitis in Gorakhpur district, Uttar Pradesh. Indian J Med Res. 2003 Mar;117:104–10.

21. Kanojia PC, Geevarghese G. New mosquito records of an area known for Japanese encephalitis hyperendemicity, Gorakhpur District, Uttar Pradesh, India. J Am Mosq Control Assoc. 2005 Mar;21(1):1–4.

22. Misra BR, Gore M. Malathion Resistance Status and Mutations in Acetylcholinesterase Gene (Ace) in Japanese Encephalitis and Filariasis Vectors from Endemic Area in India. J Med Entomol. 2015 May;52(3):442–6.

23. Rajavel AR, Natarajan R, Vaidyanathan K. Mosquitoes of the mangrove forests of India: Part 2--Sundarbans, West Bengal. J Am Mosq Control Assoc. 2005 Jun;21(2):136–8.

24. Mariappan T, Samuel P, Thenmozhi V, Paramasivan R, Sharma P, Biswas A, et al. Entomological investigations into an epidemic of Japanese encephalitis (JE) in northern districts of West Bengal, India (2011-2012). Indian J Med Res. 2014 Nov;139(5):754–61.
