# Supplementary material for: Joint spatiotemporal modelling reveals seasonally dynamic patterns of Japanese encephalitis vector abundance across India
Source: PLoS Negl Trop Dis. 2022 Feb 22;16(2):e0010218. doi: 10.1371/journal.pntd.0010218 (PMC8896663; doi:10.1371/journal.pntd.0010218)
Supplement: S2 Table — The table includes the sources and rationale (hypothesises) for inclusion of covariates in spatiotemporal models of vector abundance. (DOCX) [file pntd.0010218.s007.docx]

**S2 Table. Data and rationale for covariates included in analyses.** The table includes the sources and rationale (hypothesises) for inclusion of covariates in spatiotemporal models of vector abundance.

| **Covariate** | **Dataset** | **Description** | **Spatial resolution** | **Temporal resolution** | **Data classification** | **Source** | **Rationale** |
| --- | --- | --- | --- | --- | --- | --- | --- |
| Mean, min and max air temperature | TerraClimate | High-spatial resolution data WorldClim is combined with coarser spatial resolution, but time-varying data from CRU Ts4.0 and JRA55. | 1/24°,  ~4 km; Global. | Monthly:  1958–2019. | Maximum temperature, minimum temperature, and derived mean temperature (⁰C). | <http://www.climatologylab.org/terraclimate.html> | Temperature affects important vector life history traits such as development rate and survival [1]. |
| Mean precipitation | TerraClimate | High-spatial resolution data WorldClim is combined with coarser spatial resolution, but time-varying data from CRU Ts4.0 and JRA55. | 1/24°,  ~4 km; Global. | Monthly:  1958–2019. | Precipitation (mm). | <http://www.climatologylab.org/terraclimate.html> | Rainfall has been shown to influence vector populations due to the creation of standing water for vector breeding [2–4]. |
| Land cover | European Space Agency Climate Change Initiative (CCI) Land Cover; version 3.14. | Land cover time series produced with the reprocessing and the interpretation of five different satellite missions providing daily observation of the Earth. | 300m,  Global. | Annual:  1992 -2015. | 37 UN Land Cover Classes, derived into six broad groups: agricultural, mixed agricultural, forest, mixed vegetation, urban and water. | <http://maps.elie.ucl.ac.be/CCI/viewer/index.php> | Irrigated agricultural practices provide suitable habitat for vector development and *C. tritaeniorhynchus* is reported to preferentially breed in rice paddy fields [5,6]. |
| Land use intensity metrics for rice crop cultivation | RiceAtlas;  version 2. | Database of rice planting and harvesting dates by growing season and estimates of monthly production for all rice-producing countries. | Second level subdivisions (i.e., district-level for India),  Global. | 2010–2012 average. | Location information – geographic scale / crop calendar -planting, harvesting, growing / production / area. | [7] | Vector abundance is positively associated with rice field density [8], rice crop growth stage [9,10] and standing water availability [5,11]. |

**References**

1. Mordecai EA, Caldwell JM, Grossman MK, Lippi CA, Johnson LR, Neira M, et al. Thermal biology of mosquito-borne disease. Ecol Lett. 2019 Oct;22(10):1690–708.

2. Reisen W, Aslamkhan M, Basia R. The effects of climatic patterns and agricultural practices on the population dynamics of Culex tritaeniorhynchus in Asia. Southeast Asian J Trop Med Public Health. 1976;7(61–71).

3. Murty US, Rao MS, Arunachalam N. The effects of climatic factors on the distribution and abundance of Japanese encephalitis vectors in Kurnool district of Andhra Pradesh, India. J Vector Borne Dis. 2010;(47):26–32.

4. Vythilingam I, Oda K, Mahadevan S, Abdullah G, Thim CS, Hong CC, et al. Abundance, parity, and Japanese encephalitis virus infection of mosquitoes (Diptera:Culicidae) in Sepang District, Malaysia. J Med Entomol. 1997;34(3):257–62.

5. Keiser J, Maltese MF, Erlanger TE, Bos R, Tanner M, Singer BH, et al. Effect of irrigated rice agriculture on Japanese encephalitis, including challenges and opportunities for integrated vector management. Acta Trop. 2005;95(1):40–57.

6. Sabesan S, Raju Konuganti HK, Perumal V. Spatial Delimitation, Forecasting and Control of Japanese Encephalitis: India - A Case Study. Open Parasitol J. 2008 Sep 25;2(1):59–63.

7. Laborte AG, Gutierrez MA, Balanza JG, Saito K, Zwart SJ, Boschetti M, et al. Data Descriptor: RiceAtlas, a spatial database of global rice calendars and production. Sci Data. 2017;4:1–10.

8. Richards EE, Masuoka P, Brett-Major D, Smith M, Klein TA, Kim HC, et al. The relationship between mosquito abundance and rice field density in the Republic of Korea. Int J Health Geogr. 2010 Jun;9(1):32.

9. Raju HK, Sabesan S, Rajavel AR, Subramanian S, Natarajan R, Thenmozhi V, et al. A preliminary study to forecast Japanese encephalitis vector abundance in paddy growing area, with the aid of radar satellite images. Vector-Borne Zoonotic Dis. 2016;16(2):117–23.

10. Raju HK, Sabesan S, Subramanian S, Jambulingam P. Validating the association of Japanese encephalitis vector abundance with paddy growth, using MODIS data. Vector-Borne Zoonotic Dis. 2018;18(10):560–2.

11. Rajagopalan PK, Panicker KN. A note on the 1976 epidemic of Japanese encephalitis in Burdwan district, West Bengal. Indian J Med Res. 1978 Sep;68:3938.
