# Supplementary material for: Joint spatiotemporal modelling reveals seasonally dynamic patterns of Japanese encephalitis vector abundance across India
Source: PLoS Negl Trop Dis. 2022 Feb 22;16(2):e0010218. doi: 10.1371/journal.pntd.0010218 (PMC8896663; doi:10.1371/journal.pntd.0010218)
Supplement: S3 Table — The table details the structure of the joint-likelihood models and the difference between their corresponding within-sample predictive accuracy assessed on Watanabe-Akaike Information Criterion (WAIC) values when additional absence data are excluded. The differences (Δ) in WAIC from the baseline for the environmental and seasonal models are still equivalently large when compared to the ΔWAIC values when the additional absence data are included. (DOCX) [file pntd.0010218.s008.docx]

**S3 Table. Impact of additional inferred absence data on selection results for models of increasing complexity.** The table details the structure of the joint-likelihood models and the difference between their corresponding within-sample predictive accuracy assessed on Watanabe-Akaike Information Criterion (WAIC) values when additional absence data is excluded. The differences ($\Delta$) in WAIC between the other models and the best fitting environmental model are still equivalently large when compared to the $\Delta$WAIC values when the additional absence data is included.

| Model | | Fixed effects | Random intercepts | WAIC | $\boldsymbol{\Delta}$WAIC | $\boldsymbol{\Delta}$WAIC for model with additional absence data |
| --- | --- | --- | --- | --- | --- | --- |
| 1 | Baseline model | - | ST, S | 721.60 | 72.94 | 77.53 |
| 2 | Seasonal model | - | ST, S, M | 652.62 | 3.96 | 6.52 |
| 3 | Environmental model | Precipitation,  Agri. land proportion,  Annual rice crops,  Annual rice area,  Annual rice production,  Nonlinear temp. function | ST, S, M | 648.66 | 0.00 | 0.00 |
