# Supplementary material for: Joint spatiotemporal modelling reveals seasonally dynamic patterns of Japanese encephalitis vector abundance across India
Source: PLoS Negl Trop Dis. 2022 Feb 22;16(2):e0010218. doi: 10.1371/journal.pntd.0010218 (PMC8896663; doi:10.1371/journal.pntd.0010218)
Supplement: S4 Table. Model comparison results for observed JE outbreaks. AIC, odds ratio and 95% confidence intervals reported from logistic regression of JE outbreak probability as a function of model predicted vector abundance. Vector abundance predictions were generated from the final model with and without — (DOCX) [file pntd.0010218.s009.docx]

**S4 Table. Model comparison results for observed JE outbreaks.** AIC, the difference in AIC between the models and the best fitting model ($\Delta$AIC), the Akaike weight, odds ratio and 95% confidence intervals reported from logistic regression of JE outbreak probability as a function of model predicted vector abundance. Vector abundance predictions were generated from the final model with and without a one-month lag. A null model (i.e., intercept only) was developed to assess the ability of vector abundance predictions in estimating JE outbreaks when compared to predictions expected at random.

| **Model** | **AIC** | $\Delta$**AIC** | **Akaike weight** | **Odds ratio** | **95% Confidence interval** |
| --- | --- | --- | --- | --- | --- |
| Null (intercept-only) | 168.02 | 23.85 | 0 | - | - |
| No lag  (JE outbreak probability as a function of predicted vector abundance in the same month) | 147.66 | 3.49 | 0 | 2.25 | 1.35 - 3.74 |
| One month lag  (JE outbreak probability as a function of predicted vector abundance in the previous month) | 144.17 | 0.00 | 1 | 2.45 | 1.52 - 4.08 |
