# Supplementary material for: Joint spatiotemporal modelling reveals seasonally dynamic patterns of Japanese encephalitis vector abundance across India
Source: PLoS Negl Trop Dis. 2022 Feb 22;16(2):e0010218. doi: 10.1371/journal.pntd.0010218 (PMC8896663; doi:10.1371/journal.pntd.0010218)
Supplement: Attachment — Submitted filename: ReviewComments.docx [file pntd.0010218.s010.docx]

This study used published data on *Culex tritaeniorhynchus* presence and abundance in India to: i) quantify the relative importance of environmental drivers of abundance; ii) produce seasonal maps of Japanese encephalitis (JE) hazard; and iii) determine whether vector abundance estimates are associated with JE cases. The authors conclude that patterns of vector abundance were driven by seasonality and environmental factors and model predictions of vector abundance could be used as a proxy for JE hazard across India.

The findings of this paper with respect to aim i) and iii) are largely in accord with previous studies, including those used as a source of data in this work and therefore not particularly original. It is well established that primary JEV vector species have larval habitats in rice paddies and population dynamics are affected by monsoon rains in addition to irrigation. It is also already well demonstrated that JEV transmission dynamics and therefore JE cases are in part driven by vector abundance. While I think this is not necessarily a big problem in terms of publication, there are additional concerns with the manuscript that I would suggest need to be resolved before publication. It is clear substantial effort has gone into data collection, curation and analysis.

1. **Conclusion that the predictions of vector abundance could be used as a proxy for JE hazard.**

I don’t agree that the resulting maps are of JE hazard, they are of *Cx. tritaeniorhynchus* predicted abundance. The conclusion that the predictions can be used as a proxy for JE hazard are not justified by the evidence presented. Vector abundance is a risk factor that contributes to hazard, but does not, by itself determine infection risk. Importantly, the model only focuses on one JEV vector species and does not account for the dynamics of other competent vector species likely to contribute to risk of becoming infected with JEV in any time or place. The model also does not account for other factors driving transmission dynamics that determine JE hazard.

Why not just simplify the paper by referring throughout to predicted abundance rather than hazard? With respect to the use of ‘hazard’, also see point 4 in relation to manuscript framing and terminology.

2. **Ability to predict beyond areas where data are collected based on environmental covariates.**
a) More detail on the data collected needs to be provided in the main method section. Can you provide a summary table with each row a study stating how many data points from that study, method(s) of trapping, year from and to etc? It needs to be explicitly stated that most of the data are not only from Tamil Nadu but also from a single study.
b) What is the MAE without random effects of study and state-level spatial random effects? E.g. how much variation is explained by these random effects relative to the variation from environmental covariates that you are proposing could be used to predict vector abundance in new sites? From looking at Fig S2 I am concerned that actually the environmental covariates are not very informative with respect to explaining variation in vector abundance once random effect of study is accounted for. I think this should be toned down in the results and discussion and clearly acknowledged… is the model all that good?!
c) Acknowledge the limitations of your assessment of predictive ability. Most of the data are from Tamil Nadu and therefore most of the out of sample predictions will also be from there? Not from areas where people likely interested to understand predictive ability that haven’t been subject to survey where the model is likely a lot less accurate and/ or precise?

d) Fig 2. Uncertainty should be presented as separate map layers, as is it’s hard to interpret.

3. **Is the analysis correlating JE cases with vector abundance appropriate and can presentation of results be improved?**

*Methods*

a) The section on lines 408 – 419 is confusing. On line 413 what is JE outbreak probability? How was this calculated? How is an outbreak defined? In India JE is endemic and, in some locations, can occur all year round.
b) The locations where vectors are studied are biased toward areas previously reported JE cases and where vector abundance is high, so this analysis seems circular to me. If pseudoabsence locations were generated elsewhere these are of course likely to be places where many JE cases haven’t been recorded?! It therefore doesn’t surprise me that model predictions of abundance do correlate with JE cases as these studies were done in locations where JE occurs? The study where many of the data points are from (Samuel et al 2016) is a case in point.

I don’t have a suggested fix for the method limitation other than removing or acknowledging in the discussion if it is retained and toning down the usefulness of the model for a proxy of JE hazard.

*Results*

c) Please refer to Burnham and Anderson (2002) for best presentation of results using information criteria – i.e. in table include likelihood, AIC, the difference in AIC between the models and the best fitting model and the Akaike weights. While this is useful for determining the best model from a set reflecting competing hypotheses, I worry here it’s not particularly useful in determining how much variation in JE case occurrence can be attributed to variation in *Cx. tritaeniorhynchus* abundance? Of course, from the odds ratio yes, I can see there is an increased risk – as would be expected – but for areas not subject to study, how good is the model at predicting JE occurrence? This cannot be quantified as is. Thus, the conclusion that abundance can be used as a proxy for hazard isn’t justified.
d) Is there a way you can show JE case data and predictions graphically? Do they correlate within the areas where you have data at all (e.g. without incl. pseudo data)? I think this question stems from not quite understanding what the dependent variable is (2a).
e) Caution should be taken and clearly acknowledged when extrapolating at a national scale from scant village or between-village level data. See Ploton et al 2020 <https://www.nature.com/articles/s41467-020-18321-y> I think in the least this should be addressed in the discussion. I think it would be reasonable instead in the discussion to emphasise potentially using the predictions to guide surveillance efforts to test properly the model ability to predict abundance and JE hazard.

Minor comments

*Multiple areas in manuscript*. The term ‘hazard’ is first used on line 81 ‘MBD risk can be understood as the likelihood of an outbreak due to exposure of a susceptible population to an infected mosquito vector (hazard) (ref 5).

As suggested in 1, would consider removing term ‘hazard’ and simplifying to what the predictions are – of abundance. If you are going to use ‘hazard’ think it might be good reconsider definition(s) in the referenced study here (5): ‘*As in the field of quantitative risk analysis [14], a hazard is a potential source of harm, while risk is the likelihood of adverse events (e.g. disease outbreaks) caused by exposure to a hazard (e.g. pathogen), potentially weighted by the severity of the adverse event (e.g. expexcted number of cases).*’

In this context is Cx. t. a risk factor for JE rather than JE hazard? This terminology makes clearer that there will be other risk factors that are important to consider. Following this definition in the cited paper reads:

For example, in the case of Ebola virus disease, Ebola viruses circulating in wildlife are the hazard, an outbreak in the human population is the adverse event, and there are a number of risk factors, bushmeat consumption, hunting in the deep forest and quality of public health infrastructure, that can affect the risk of an outbreak by influencing its probability or severity [15].

So here the parallel would be JEV viruses circulating in the wild/ livestock population and mosquitoes?

These terms and nuances are making it difficult to see the wood for the trees – why not stick to talking about *Cx. t.* abundance and predicting this throughout the manuscript – with a one sentence or two in the intro and discussion that, of course, as is well documented, abundance/ density is a critical driver in mbd dynamics and therefore contributes to hazard?

*Introduction*

80 Don’t start sentence with an abbreviation

83 Would argue these don’t estimate hazard, but a component of determining risk – the circulating pathogen is the hazard.

88 A lot of parentheses interrupts reading re-write without using parentheses

89-91 Not sure necessarily correct for JEV here – high abundance year-round likely results in endemic transmission in the reservoir host populations and may could potentially result in lower cases of human infections as don’t get a seasonal large epidemic resulting from a build-up of susceptible individuals in the reservoir population. I could be wrong but point is to consider wider context and dynamics not just vector abundance. Important here to consider how livestock production systems together with vector dynamics impact risk.

95-96 What do you mean by ‘changes in their contact’? This is too vague.

124-125 See more recent work from Quan et al <https://www.ncbi.nlm.nih.gov/pmc/articles/PMC7282807/>

*Methods*

185 How was the literature search carried out? Was this a systematic review of the literature done? More detail required here.

195 ‘High resolution’ environmental variables? At what resolution?

204 Inappropriate use of the term ‘density’ – this should be abundance?

213 Additional inferred absence data

Known absence of mosquitoes above 2000m – reference given is from >10 yrs ago & I can’t see Cx. t. recorded in this paper even at lower altitudes? This may be due to absence of suitable habitats. This isn’t sufficient to justify this part of the methods. See:

<https://www.jvbd.org/article.asp?issn=0972-9062;year=2017;volume=54;issue=1;spage=69;epage=73;aulast=Zhang>

<https://www.ncbi.nlm.nih.gov/pmc/articles/PMC2866384/>

If this approach is retained, needs to at least be highlighted and discussed with respect to climate change.

291 ‘from 2009’ makes it sound like only data were obtained for 2009.

*Results*

422 Rename to something more biologically meaningful than ‘model selection’

288 – ‘revealed shifting locations of high or low Cxt abundance between seasons’ rephrase wording is confusing.

*Discussion*

542 – 547 as suggested above, might want to shift emphasis here to guiding surveillance rather than intervention

549 – 566 Can you state what new findings this presents that wasn’t concluded from the individual studies the raw data are from?

568 – 590 As per 549 - 566

581 Consider whether these areas have fewer hosts?

603-606 Don’t quite follow. ‘*Predicted hotspots in the southeast did not correspond to high cases, potentially revealing the importance of vertical transmission for this disease (28) which requires consistently high vector abundance to maintain the virus in the vector population*.’ This makes it sound as though vertical transmission requires high vector abundance, but the opposite is true – vertical transmission for some arboviruses can enable persistence during times of low adult abundance.

Why is national vaccination programme not referred to here, or absence of reservoir hosts, or underreporting due to lack of hospital access, or the many other reasons why abundance doesn’t directly indicate hazard – defined as the circulating pathogen?!
