# Supplementary material for: Joint spatiotemporal modelling reveals seasonally dynamic patterns of Japanese encephalitis vector abundance across India
Source: PLoS Negl Trop Dis. 2022 Feb 22;16(2):e0010218. doi: 10.1371/journal.pntd.0010218 (PMC8896663; doi:10.1371/journal.pntd.0010218)
Supplement: Attachment — Submitted filename: Reviewer Responses_271021.docx [file pntd.0010218.s011.docx]

**Reviews of Manuscript No.:** PNTD-D-21-00888

**Title:** Joint spatiotemporal vector modelling reveals seasonally dynamic hazard patterns of Japanese encephalitis across India.

**Authors:** Lydia H.V. Franklinos, David W. Redding, Tim C.D. Lucas, Rory Gibb, Ibrahim Abubakar, Kate E. Jones.

We thank the editors and the two reviewers for taking the time to review our work and for their positive feedback and helpful suggestions for improving our manuscript. Please find below our responses to the queries raised, shown in red, with reference to specific line numbers or figure/tables. All line numbers refer to the marked-up copy version of the revised manuscript.

**Reviewer #1:**

This study used published data on Culex tritaeniorhynchus presence and abundance in India to: i) quantify the relative importance of environmental drivers of abundance; ii) produce seasonal maps of Japanese encephalitis (JE) hazard; and iii) determine whether vector abundance estimates are associated with JE cases. The authors conclude that patterns of vector abundance were driven by seasonality and environmental factors and model predictions of vector abundance could be used as a proxy for JE hazard across India.

The findings of this paper with respect to aim i) and iii) are largely in accord with previous studies, including those used as a source of data in this work and therefore not particularly original. It is well established that primary JEV vector species have larval habitats in rice paddies and population dynamics are affected by monsoon rains in addition to irrigation. It is also already well demonstrated that JEV transmission dynamics and therefore JE cases are in part driven by vector abundance. While I think this is not necessarily a big problem in terms of publication, there are additional concerns with the manuscript that I would suggest need to be resolved before publication. It is clear substantial effort has gone into data collection, curation and analysis.

Response - Thank you for your thorough review and for recognising the merits of our study.

Our study provides a novel method to leverage information from sparse vector surveillance data and predict vector abundance for the predominant vector of Japanese encephalitis in India. As concluded by Reviewer 2, this approach will have wide applications for other neglected vector-borne disease systems where vector surveillance efforts are patchy. Our research enabled the presentation of seasonal vector abundance at a national scale, addressing the previous lack of knowledge on the spatiotemporal distribution of this vector species across India and the importance of environmental factors in driving broad-scale spatiotemporal patterns of JE vector abundance.

We believe we have addressed your concerns including changing the way we relate vector abundance to the term ‘hazard’, providing further information on the mosquito surveillance data, clarifying the model development process and predictive ability of the model, and reframing our analysis correlating JE cases with vector abundance. We believe that these corrections have enhanced the manuscript.

1. **Conclusion that the predictions of vector abundance could be used as a proxy for JE hazard.**

I don’t agree that the resulting maps are of JE hazard, they are of Cx. tritaeniorhynchus predicted abundance. The conclusion that the predictions can be used as a proxy for JE hazard are not justified by the evidence presented. Vector abundance is a risk factor that contributes to hazard, but does not, by itself determine infection risk. Importantly, the model only focuses on one JEV vector species and does not account for the dynamics of other competent vector species likely to contribute to risk of becoming infected with JEV in any time or place. The model also does not account for other factors driving transmission dynamics that determine JE hazard.

Why not just simplify the paper by referring throughout to predicted abundance rather than hazard? With respect to the use of ‘hazard’, also see point 4 in relation to manuscript framing and terminology.

Response - The hazard is the pathogen (JE virus) in mosquito vectors since this is the form in which the pathogen resembles a source of harm to humans. JE hazard requires the presence of reservoir hosts with the pathogen and mosquito vectors which become infected. Therefore, vector abundance is a key component of JE hazard since vectors are required to transmit the pathogen and so the abundance of vectors will influence the hazard.

However, we do agree that statements throughout the manuscript describing vector abundance as a proxy for MBD/JE hazard are too strong and so we have changed the text throughout to describe vector abundance as a *component* of hazard (see lines 59, 90, 153, 191). We have also added a sentence explaining that to define hazard would require information on pathogen prevalence in the host and vector populations (see lines 87-9).

We agree that the maps should be described as vector abundance rather than JE hazard and have changed them and the text accordingly in the method, results and discussion. On reflection, we have also decided that the third aim of the paper “to test whether mosquito abundance estimations are strongly associated with JE cases and therefore are a good proxy for JE hazard” is not achieved since the analysis we have provided is relatively crude. Instead, we have decided to use the summary statistic provided by our analysis as a discussion point on the potential for vector abundance as a proxy for hazard which we believe has allowed us to include some of the critique that you have provided (see lines 195-9, 720-42). We have edited the abstract and author summary to reflect these changes.

2. **Ability to predict beyond areas where data are collected based on environmental covariates.**
a) More detail on the data collected needs to be provided in the main method section. Can you provide a summary table with each row a study stating how many data points from that study, method(s) of trapping, year from and to etc? It needs to be explicitly stated that most of the data are not only from Tamil Nadu but also from a single study.

Response - We have now provided an additional supplementary data table outlining this information and have referred to it in the text (see S1 Table).

b) What is the MAE without random effects of study and state-level spatial random effects? E.g. how much variation is explained by these random effects relative to the variation from environmental covariates that you are proposing could be used to predict vector abundance in new sites? From looking at Fig S2 I am concerned that actually the environmental covariates are not very informative with respect to explaining variation in vector abundance once random effect of study is accounted for. I think this should be toned down in the results and discussion and clearly acknowledged… is the model all that good?!

Response – Both the study and state-level spatial random effects are critical to the modelling framework and so testing for MAE without them would introduce additional noise due to confounding and prevent clear interpretation of the relative influence of environmental covariates on vector abundance. The state-level (and seasonal) effects are an integral part of the predictive model, providing a spatiotemporal dependency structure for prediction (Lowe et al., 2021) and should be considered as such (and we consider in the discussion the need to collect more data to understand what else is driving these broad patterns). A study-level random effect is necessary to remove confounding effects of study (e.g., unobserved variables such as study-level differences in surveillance methods) to get unbiased estimates of key parameters. For clarity, we have made the distinction between these effects clearer in the manuscript by removing the term “random” from spatial and seasonal effects and just using it to describe the study-level effects (see Model specification section in the Methods and changes throughout manuscript).

MAE was used to calculate the difference between the predicted posterior mean values and the corresponding observed values of vector abundance as a measure of model predictive ability. As described in Table 1, the environmental model further reduced out-of-sample MAE by 40% relative to baseline+seasonal model so the inclusion of environmental covariates is providing a substantial improvement that is much larger than described in other models applying a similar analytical framework (e.g., ~ 8% in out-of-sample RMSE for dengue forecasting in Thailand; Lowe et al. 2016).

c) Acknowledge the limitations of your assessment of predictive ability. Most of the data are from Tamil Nadu and therefore most of the out of sample predictions will also be from there? Not from areas where people likely interested to understand predictive ability that haven’t been subject to survey where the model is likely a lot less accurate and/ or precise?

Response - We acknowledge the limitations in the predictive ability of the model by mapping uncertainty in model predictions in Fig 3. We also highlight this in the discussion section (see lines 746-8) and state that more surveillance data are needed to improve predictions. We have now added some more information in the limitation section of the discussion to clarify this point (see lines 748-58).

d) Fig 2. Uncertainty should be presented as separate map layers, as is it’s hard to interpret.

Response - We believe you are referring to Fig 3 which presents predicted seasonal abundance of *C. tritaeniorhynchus* across India with uncertainty represented in the maps by transparency. We believe that the current version of Fig 3 is much easier to understand than having two separate maps to read and reference across per season as suggested here by reviewer 1. We believe side-by-side maps also have significant barriers to interpretation due to issues with having to geographically link the different data they contain by eye rather than viewing the different data simultaneously mapped onto the same location.

3. **Is the analysis correlating JE cases with vector abundance appropriate and can presentation of results be improved?**

*Methods*

a) The section on lines 408 – 419 is confusing. On line 413 what is JE outbreak probability? How was this calculated? How is an outbreak defined? In India JE is endemic and, in some locations, can occur all year round.

Response – We have changed the text to define JE outbreak probability as “the probability that an outbreak occurred”. We have now provided a definition for a JE outbreak as one or more confirmed or suspects cases of JE occurring in the same village within the same month. JE outbreak probability was calculated as a binomial (presence/absence) probability that a JE outbreak occurred in a particular geographic location and calendar month. We have edited this section to clarify the terms and method (see lines 488-98).

b) The locations where vectors are studied are biased toward areas previously reported JE cases and where vector abundance is high, so this analysis seems circular to me. If pseudoabsence locations were generated elsewhere these are of course likely to be places where many JE cases haven’t been recorded?! It therefore doesn’t surprise me that model predictions of abundance do correlate with JE cases as these studies were done in locations where JE occurs? The study where many of the data points are from (Samuel et al 2016) is a case in point.

I don’t have a suggested fix for the method limitation other than removing or acknowledging in the discussion if it is retained and toning down the usefulness of the model for a proxy of JE hazard.

Response – For our predictive model of vector abundance, we found that despite abundance predictions being relatively focal (Fig 2), the spatiotemporal distribution of vector sampling in the data was more evenly distributed across India (Fig 1). This suggests that spatial bias is not driving our model predictions (see lines 758-61). In addition, as highlighted in our discussion, high vector abundance (a component of JE hazard) does not necessarily confer JE outbreaks (JE risk) since additional factors that influence the hazard (e.g., data on animal reservoir host populations), potential exposure (e.g., human demography, socioeconomics) and susceptibility (e.g., vaccination coverage) of human populations need to be considered. Indeed, predicted seasonal hotspots of vector abundance in the southeast (where most data are collected) did not correspond to high recorded cases (Fig 3), potentially reflecting other factors such as vaccine coverage that are preventing outbreaks.

*Results*

c) Please refer to Burnham and Anderson (2002) for best presentation of results using information criteria – i.e. in table include likelihood, AIC, the difference in AIC between the models and the best fitting model and the Akaike weights. While this is useful for determining the best model from a set reflecting competing hypotheses, I worry here it’s not particularly useful in determining how much variation in JE case occurrence can be attributed to variation in Cx. tritaeniorhynchus abundance? Of course, from the odds ratio yes, I can see there is an increased risk – as would be expected – but for areas not subject to study, how good is the model at predicting JE occurrence? This cannot be quantified as is. Thus, the conclusion that abundance can be used as a proxy for hazard isn’t justified.

Response – We have adjusted the presentation of results in S4 Table to include the delta AIC and Akaike weights as suggested in Burnham and Anderson (2002).

We agree with your comments about our conclusion that abundance can be used as a proxy for hazard. On reflection, we have decided that the third aim of the paper “to test whether mosquito abundance estimations are strongly associated with JE cases and therefore are a good proxy for JE hazard” is not achieved since the analysis we have provided is relatively crude, as you have highlighted. Instead, we have decided to use the summary statistic provided by our simple correlative analysis as a discussion point on the potential for using vector abundance as a proxy for hazard (see lines 195-9, 720-42). We have explained in the discussion that to fully gauge the strength of this association and assess the usefulness of vector abundance as potential proxy for JE hazard would require a more complex model that accounts for temporal and spatial autocorrelation in model residuals and uncertainty in the model (see lines 724-8).

d) Is there a way you can show JE case data and predictions graphically? Do they correlate within the areas where you have data at all (e.g. without incl. pseudo data)? I think this question stems from not quite understanding what the dependent variable is (2a).

Response – We present vector abundance predictions and JE case data in Fig 3 in which the black circles represent the location and magnitude (i.e., number of cases) for JE human outbreaks per season during the period 2009-2015 across India. Case data correlates with vector abundance data (Fig 1) in the north and north-eastern regions of India however, predicted seasonal hotspots of vector abundance in the southeast (where most data are collected) did not correspond to a high number of reported cases (Fig 3). As described previously, we have decided to use the summary statistic provided by our simple correlative analysis as a discussion point on the potential for using vector abundance as a proxy for hazard (see lines 195-9, 720-42) since we believe our relatively crude analysis does not answer whether mosquito abundance estimations can be used as a proxy for JE hazard.

We have explained in the discussion that to fully gauge the strength of this association would require a more complex model that accounts for temporal and spatial autocorrelation in model residuals and uncertainty in the model (see lines 724-8).

e) Caution should be taken and clearly acknowledged when extrapolating at a national scale from scant village or between-village level data. See Ploton et al 2020 <https://www.nature.com/articles/s41467-020-18321-y> I think in the least this should be addressed in the discussion. I think it would be reasonable instead in the discussion to emphasise potentially using the predictions to guide surveillance efforts to test properly the model ability to predict abundance and JE hazard.

Response – Our understanding of the Ploton et al. 2020 paper is that it shows that a model that appears highly predictive becomes much less so when spatial autocorrelation is accounted for. We accounted for spatial autocorrelation in the methods, and it forms a major component of the model to prevent overoptimistic assessment of model predictive power as described in Ploton et al.

We do agree however that model predictions will not provide accurate data at the local level, instead they reveal broad scale ecological patterns that can help to direct future research efforts. We have now edited our discussion to acknowledge this and emphasise the use of our work to guide surveillance efforts and validate our results (see lines 749-54, 774-90).

Minor comments

*Multiple areas in manuscript.*

The term ‘hazard’ is first used on line 81 ‘MBD risk can be understood as the likelihood of an outbreak due to exposure of a susceptible population to an infected mosquito vector (hazard) (ref 5). As suggested in 1, would consider removing term ‘hazard’ and simplifying to what the predictions are – of abundance. If you are going to use ‘hazard’ think it might be good reconsider definition(s) in the referenced study here (5): ‘As in the field of quantitative risk analysis [14], a hazard is a potential source of harm, while risk is the likelihood of adverse events (e.g. disease outbreaks) caused by exposure to a hazard (e.g. pathogen), potentially weighted by the severity of the adverse event (e.g. expected number of cases).’

Response – In the definition provided by the referenced study (5), a hazard is the “potential source of harm” which refers to a pathogen that is transmissible to a human. For mosquito-borne diseases the hazard is the pathogen in mosquito vectors since this is the form in which the pathogen resembles a source of harm to humans. Therefore, vector abundance is a key component of MBD hazard since vectors are required to transmit the pathogen and so the abundance of vectors will influence the hazard.

However, we agree that statements throughout the manuscript describing vector abundance as a proxy for JE hazard are too strong and so we have changed the text throughout to describe vector abundance as a *component* of hazard (see lines 59, 90, 153, 191). We also agree that the predictions should be described as vector abundance rather than JE hazard and have changed the text accordingly in the method, results and discussion.

In this context is Cx. t. a risk factor for JE rather than JE hazard? This terminology makes clearer that there will be other risk factors that are important to consider. Following this definition in the cited paper reads: For example, in the case of Ebola virus disease, Ebola viruses circulating in wildlife are the hazard, an outbreak in the human population is the adverse event, and there are a number of risk factors, bushmeat consumption, hunting in the deep forest and quality of public health infrastructure, that can affect the risk of an outbreak by influencing its probability or severity [15]. So here the parallel would be JEV viruses circulating in the wild/ livestock population and mosquitoes?

Response – For Ebola virus disease, the hazard is the pathogen in reservoir hosts since transmission occurs from wildlife-human directly without a vector. As described in the Ebola virus example, a risk factor relates to the likelihood of exposure to the hazard i.e., the consumption of bushmeat, hunting etc. Comparatively, for JE the hazard is a mosquito vector infected with JE virus. This requires the pathogen in reservoir hosts and the presence of mosquito vectors which become infected when biting the hosts. Therefore, vector abundance is a key component of JE hazard since vectors are required to transmit the pathogen to humans and so the abundance of vectors will influence the hazard. As you have stated, several other vectors in addition to *C. tritaeniorhynchus* will also constitute the hazard. Nevertheless, as the predominant vector for the transmission of JE in Asia we still believe that the abundance of this vector will be a key component of JE hazard. To clarify the terminology, we have added a sentence explaining that to define hazard would require information on pathogen prevalence in the host and vector populations (see lines 87-9).

These terms and nuances are making it difficult to see the wood for the trees – why not stick to talking about Cx. t. abundance and predicting this throughout the manuscript – with a one sentence or two in the intro and discussion that, of course, as is well documented, abundance/ density is a critical driver in mbd dynamics and therefore contributes to hazard?

Response – We agree that statements throughout the manuscript describing vector abundance as a proxy for MBD/JE hazard are too strong and so we have changed the text throughout to describe vector abundance as a *component* of hazard (see lines 59, 90, 153, 191). We also agree that the predictions should be described as vector abundance rather than JE hazard and have changed the text accordingly in the method, results, and discussion.

*Introduction*

80 Don’t start sentence with an abbreviation

Response – We have made the suggested edit.

83 Would argue these don’t estimate hazard, but a component of determining risk – the circulating pathogen is the hazard.

Response – We agree that statements throughout the manuscript describing vector abundance as a proxy for MBD/JE hazard are too strong and so we have changed the text throughout to describe vector abundance as a *component* of hazard. Please see a more detailed answer to this question in our response to Q1.

88 A lot of parentheses interrupts reading re-write without using parentheses

Response – Done.

89-91 Not sure necessarily correct for JEV here – high abundance year-round likely results in endemic transmission in the reservoir host populations and could potentially result in lower cases of human infections as don’t get a seasonal large epidemic resulting from a build-up of susceptible individuals in the reservoir population. I could be wrong but point is to consider wider context and dynamics not just vector abundance. Important here to consider how livestock production systems together with vector dynamics impact risk.

Response – We agree that this sentence is too strongly worded regarding high perennial vector abundance influencing hazard. Therefore, we have edited the sentence to clarify that it instead modifies factors that influence MBD hazard such as pathogen establishment and persistence (see lines 100-3). We have also added a sentence explaining that to define hazard would require information on pathogen prevalence in the host and vector populations (see lines 87-9).

95-96 What do you mean by ‘changes in their contact’? This is too vague.

Response – We have added “...that could lead to pathogen exposure i.e., via biting” to be more specific about how the contact is associated with pathogen transmission.

124-125 See more recent work from Quan et al <https://www.ncbi.nlm.nih.gov/pmc/articles/PMC7282807/>

Response – We have now added this reference and changed the JE burden figures accordingly in lines 138-9.

*Methods*

185 How was the literature search carried out? Was this a systematic review of the literature done? More detail required here.

Response – We have now added more detail on how the literature search was conducted (see lines 210-20).

195 ‘High resolution’ environmental variables? At what resolution?

Response – We have now clarified out statement by adding details on the land cover data (see line 229-30). The resolution of the environmental variables is also provided in the methods under the title “Seasonal, environmental and land use data”.

204 Inappropriate use of the term ‘density’ – this should be abundance?

Response – We agree and have made this edit.

213 Additional inferred absence data

Known absence of mosquitoes above 2000m – reference given is from >10 yrs ago & I can’t see Cx. t. recorded in this paper even at lower altitudes? This may be due to absence of suitable habitats. This isn’t sufficient to justify this part of the methods. See:

<https://www.jvbd.org/article.asp?issn=0972-9062;year=2017;volume=54;issue=1;spage=69;epage=73;aulast=Zhang>

<https://www.ncbi.nlm.nih.gov/pmc/articles/PMC2866384/>

If this approach is retained, needs to at least be highlighted and discussed with respect to climate change.

Response – Thank you for your comment and for bringing these papers to our attention. In response to your comment, we re-ran our analysis using inferred absence vector data extracted from altitudes above 3500m since this accounts for the altitude above which *C. tritaeniorhynchus* mosquitoes have not been recorded (instead of 2000m which we incorrectly had assumed). We found that pseudoabsence data >3500m did not significantly change the model selection results or the fixed-effect parameter estimates which is not surprising given the relatively low number of datapoints (n=20). We have changed all Tables and Figures accordingly to include any changes. In addition, we have edited the method of additional inferred absence data generation and referred to the fact that climate change may mean this method would need to be reassessed in the future (see lines 749-54).

291 ‘from 2009’ makes it sound like only data were obtained for 2009.

Response – We agree and have deleted the reference to 2009 since the full date range for the data are provided in the following sentence.

*Results*

422 Rename to something more biologically meaningful than ‘model selection’

Response – We disagree that this title is inappropriate since it is describing the section of results which explains the model selection process.

488 – ‘revealed shifting locations of high or low Cxt abundance between seasons’ rephrase wording is confusing.

Response – We have reworded the sentence to make it clearer (see lines 586-8).

*Discussion*

542 – 547 as suggested above, might want to shift emphasis here to guiding surveillance rather than intervention

Response – We have made this edit (see lines 642-6).

549 – 566 Can you state what new findings this presents that wasn’t concluded from the individual studies the raw data are from?

568 – 590 As per 549 – 566

Response – Our study provides a novel method to leverage information from sparse vector surveillance data and predict vector abundance for the predominant vector of Japanese encephalitis in India. This approach will have wide applications for other neglected vector-borne disease systems where vector surveillance efforts are patchy.

Prior to this research, only a few studies had described JE vector seasonality, and those studies reported vector seasonality at local scales (see Table S1). Our research enabled the presentation of seasonal vector abundance at a national scale, addressing the previous lack of knowledge on the spatiotemporal distribution of this vector species across India. We have now added the phrase “across India” to clarify this novelty (see line 651) and have highlighted that most abundance data used in the analysis were not collected continuously (see lines 241-2) and therefore did not report seasonality.

In addition, previous studies assessing the influence of environmental predictors on vector abundance have been conducted at local scales and have not defined the importance of these factors in driving broad-scale spatial and temporal patterns of JE vector abundance. We have now edited the text to highlight this novelty (see lies 671-2, 683).

581 Consider whether these areas have fewer hosts?

Response – Since the vector can feed on multiple hosts including ardeid wading birds that use irrigated paddies as feeding habitat, it is unlikely that this would be the mechanism behind the negative association between annual area under rice cultivation and vector abundance. However, we intend to perform a future large analysis to consider this fully.

603-606 Don’t quite follow. ‘Predicted hotspots in the southeast did not correspond to high cases, potentially revealing the importance of vertical transmission for this disease (28) which requires consistently high vector abundance to maintain the virus in the vector population.’ This makes it sound as though vertical transmission requires high vector abundance, but the opposite is true – vertical transmission for some arboviruses can enable persistence during times of low adult abundance.

Why is national vaccination programme not referred to here, or absence of reservoir hosts, or underreporting due to lack of hospital access, or the many other reasons why abundance doesn’t directly indicate hazard – defined as the circulating pathogen?!

Response – We agree that this statement is confusing and so have changed it to read that seasonality in vector abundance selects for vertical transmission and added a reference to support this. We have also added other possible explanations for the described result such as unmeasured environmental factors affecting transmission, and differing vaccination and vector control measures as you have suggested (see lines 713-7).

**Reviewer #2:**

Joint likelihood modelling has been widely and successfully applied to model insect taxa to combine abundance and occurrence data or combine data at different scales to understand patterns in biodiversity. This paper represents a novel application to insect vectors and vector-borne disease epidemiology, to maximise insights from sparse occurrence and abundance data and predict hazard from mosquito vectors and risk of outbreaks, focussing on Japanese Encephalitis in India, a key neglected zoonosis there. As such it makes a great contribution to the field and will promote their wider application of these models to vector-borne disease systems and sparse vector surveillance data. The paper is very well written and motivated and should be published once revised. The statistical methods to generate the vector hazard predictions are broadly appropriate, but would benefit from some further clarification and refinement so that the reader can judge the level of support for some of the conclusions drawn. The methods for relating vector hazard to outbreaks seem rather basic and could do with some refinement to support the assertion that hazard is a valuable predictor of outbreaks. I outline these concerns in above along with some minor comments on language and clarify.

Response - Thank you for your thorough review and for noting the contribution that our work makes to the field. We believe we have addressed your concerns and hope to have clarified any issues, resulting in an improved manuscript. Specifically, we have provided further information on the mosquito surveillance data, revised and clarified the scaling of the input data and units of the mean absolute error, clarified our model selection process and reframed our analysis correlating JE cases with vector abundance.

Key concerns on statistical methods and whether they support the conclusions:

1. **Correction for mosquito sampling effort and trap types in the input data**

Line 202-205 “We calculated effort‐corrected abundance values of C. tritaeniorhynchus from the raw measurement values by aggregating monthly counts and standardising them to survey effort (one person hour) density measure for each month”

I very much appreciate the efforts to correct for sampling effort. However, further brief explanation is needed of the adult trap types and methods used. Was there any correction performed for different adult trap types (e.g. CDC versus mosquito magnets), since these are known to pull in vectors over different ranges and attract widely differing abundance levels of mosquitoes. Do you mean “one trap hour” rather than “one person hour” or are these all human landing catches?

Response - The surveillance methods varied in studies and detailed information was not available for all studies. We accounted for differences in survey methods by including study as a random effect to account for unaccounted variation in the way the vectors were sampled. We have added some text to clarify this (see line 368-71).

We agree that this information is important for future work and so have now provided an additional S1 Table that includes an overview of the studies that were used to collect the vector data and we have added information on survey method where available. We have changed the numbering of the other supplementary tables accordingly. When developing the S1 Table, we also realised that Fig 1 did not show the state of Telangana and so we have edited Fig 1 to include this.

Thank you for highlighting the issue with our description of “one person hour”. We agree that the phrase is incorrect since it does not always relate to human landing catches and so instead have changed it to “one survey hour” (see lines 239, 242).

2. **Scaling of the input data and units of the mean absolute error**

Line 335-337. It seemed strange to rescale the vector abundance data since this may obscure important biological information about hazard and size of model errors. I thought only rescaling of covariates was required to assist with assigning priors and model convergence? This is compounded later on when it is unclear whether the units for the mean absolute error (which always reflect the data) are in units of log abundance or units of the 0-1 rescaled log abundance? This makes it difficult for the reader to judge the biological significant of the difference in mean absolute error between the model with environmental factors and seasonal effects.

Response - We rescaled the predictor and explanatory variables to help with model fitting and in understanding the priors (McElreath 2020; pg 195). The continuous predictor covariates were scaled to a z (standard) score (to mean= 0, SD=1). Vector abundance was first transformed to the logscale to conform to normality and then was rescaled from 0-1 (with largest value of 1) to preserve the zero value of log vector abundance as a reference point (McElreath 2020; pg 195). We have added some text to clarify this and added a reference to support the method (see lines 382-4). We have also deleted the sentence in lines 384-6 since there is repetition from lines 242-4.

We agree that displaying the mean absolute error (MAE) in units of the 0-1 rescaled log abundance is confusing for biological interpretation, so we have decided to present MAE using the log vector abundance scale (unscaled) (see line 455 and changes to Table 1).

It would be beneficial to analyse the conditional predictive ordinates from the model and the logarithmic score based on these as an additional measure of out of fit predictive ability and to use pit histograms to check the fit of the Gaussian model to the logged abundance data.

Response - Thank you for the suggestion of including CPO and PIT values. We had incorrectly assumed that these were not needed due to complexity in their interpretation but have now added CPO and PIT plots to the supplementary materials and discussed briefly in the methods (see lines 458-65) and results (see lines 524-7 and new S3 Fig).

3. **Model selection, variable number and importance for mosquito hazard models**

Stepwise selection is used is not generally accepted as being a robust way to select subsets of covariates with full subset selection due to the risks of overfitting or identifying spurious correlations due to remaining collinearity between suites of variables. I appreciate this can be difficult with large number of predictors however and it is reassuring that a relatively small number of environmental predictors remain in the final model.

To help the reader judge if the sample size is adequate to test the effects of so many predictors with such a method, the authors should clarify the total number of environmental predictors offered to the model (following exclusion for collinearity) and how this balances out versus the sample size of month/grid square combinations (currently graphed but not specified).

Given the limited robustness of stepwise selection and to shed further light on variable importance, I would like to have seen a (supplementary info) table of the changes in WAIC when each variable is dropped from the full “environmental model” until the stopping criteria is reached. A table of environmental variables in the final model, with delta DIC values when each variable is dropped would help to give an indication of variable importance.

Response - We believe there might be some confusion about our model selection process. Rather than using stepwise selection (whereby 1. each variable is removed from full model and the delta WAIC is calculated, 2. the best of those models is used as the working model, 3. this is repeated until no candidate models are delta WAIC > 2) which we agree has limited robustness, we instead performed a single pass whereby we removed each variable from the model and calculated delta WAIC and then removed all variables where WAIC > 2. We used the single pass as a screening process to remove variables which were not improving model parsimony rather than searching for a “best subset” of variables. We have now edited the manuscript to make this clearer (see lines 427-33).

The covariates were first selected due to biological significance supported by scientific evidence (see S2 Table outlining rationale (hypothesises) for inclusion of covariates). We have clarified that we started with 17 covariates and the ended with 6 after excluding two for collinearity and excluded the others if they did not improve model parsimony by a threshold of at least 2 WAIC units (see lines 427-33). We have also now specified the number of location-months in our dataset (see line 222).

We disagree with the suggested method of evaluating the importance of the environmental variables because maximizing predictive accuracy (via improved DIC values) does not imply causation (e.g., highly confounded models can also make good predictions). In addition, the inclusion of environmental variables may be important in their interactions with other variables which cannot be evaluated with DIC comparison when each variable is dropped. Our aim is to make predictions in this study rather than infer causation which would require additional assumptions.

4. **Model linking predicted JE hazard to outbreak occurrence.**

This model seemed very simplistic given the care devoted to the mosquito hazard model. A joint likelihood or hurdle model could have been considered here for outbreak occurrence versus outbreak number. There was no correction for temporal and spatial autocorrelation in model residuals that could lead to over-estimation of vector hazard effects on outbreaks (e.g. in Lines 592-595) The accuracy may also be inflated by the large number of absence month-location combinations generated (12,000) to fit the model (though the lack of figures on the number of presence months available makes it difficult to judge the balance in the dataset). The authors should ideally test and present the sensitivity of model results to different ratios of absence to present data. Though the odds ratio for mosquito hazard seem significant (though see comments on autocorrelation), it would be helpful to give some metrics of overall variance explained in outbreak occurrence (e.g. % correct predictions) or sensitivity or specificity to help disease managers to interpret the value of the predictions (does the model do better in some seasons than another for example in this regard).

Response – Please see response to Reviewer 1’s Q1 comment.

See above comments on methods for whether the methods and results support the conclusions. The limitations of the analysis and advances in understanding of the topic are clearly described.

In terms of Public Health relevance, can you say more about practically how the maps could fit into current seasonal management of JE in India to guide which types of intervention, given the levels of uncertainty and accuracy in them and the regional variation in seasonal versus perennial occurrence?

Response - We have added some sentences on the potential use of this work (see lines 774-90).

Lines 604-606. “However, predicted seasonal hotspots in the southeast did not correspond to high cases, potentially revealing the importance of vertical transmission for this disease [28] which requires consistently high vector abundance to maintain the virus in the vector population.” This statement is a bit strong, it could also be due to unmeasured environmental factors affecting transmission or the spatial biases in the input data for the model. This should be acknowledged alongside the potential biological explanation.

Response - We agree that this statement is confusing and so have changed it to read that seasonality in vector abundance selects for vertical transmission and added a reference to support this. We have also added other possible explanations for the described result (see lines 713-20).

Line 626-627. “with the potential for future surveillance efforts to be targeted in those areas with high predicted hazard and a high degree of uncertainty (Fig 3).” I would suggest that you would also need surveillance in high predicted hazard high certainty areas rather than taking these at face value, since you have measured only some types of statistical uncertainty in your analysis and the model needs further validation with independent data.

Response - Thank you for your excellent suggestion. We agree and have edited the sentence to remove the reference to uncertainty levels and instead focus on the need for future surveillance data to validate the predictions of high vector abundance (see lines 754-8).

It would be useful to give brief information about disease impacts on communities or the size of JE burdens in the Abstract and Author Summary.

It would be useful to outline for context in the introduction where in the landscape or ecosystem human exposure to JE is thought to occur, how is this likely to be influenced by environmental or social factors that would then modulate the relationship between mosquito hazard and spill-over.

Response - Thank you for your great suggestions. We have added information about JE burdens in the abstract and author summary and have discussed human exposure in the background (see lines 141-7).

Line 109-110 “widely-used SDM approaches, such as boosted regression tree (BRT) models and MaxEnt, do not usually report metrics of predictive uncertainty, or how these vary over space [15,16].”

I think this statement is too strong. The MaxEnt model provides “clamping” metrics indicating areas that are too environmentally disimilar from the training set data to be predicted accurately. Most authors using BRTs, map variation around mean predicted probabilities across runs.

Response - We agree and have edited the text accordingly (see lines 118-21).

Line 259: With reference to JE eco-epidemiology in India, can you further explain the decision to clump classes into very broad land use types versus selecting those that are of particularly relevant to mosquito resource use.

Response - We did not have enough data to evaluate the importance of the varied land classes (i.e., Cropland, rainfed versus Cropland irrigated or post-flooding) and so broader land cover types (e.g., Cropland) were selected. We have now further expanded on this in the methods (see lines 300-3).

JE Human case data: Clarify briefly what types of location these data from IDSP encompass and how these relate to the location of hazard or exposure in the landscape and the study grain. E.g. these are primary health centre or village locations but will fall into the same 23km square in which exposure occurred.

Response - The case data were described at village level and were converted to geographic point locations (see lines 331-4). We have also highlighted the assumption that the location of vector abundance is also the location for exposure which may be inaccurate (see lines 723-8). We have also decided to just provide the result of the simple correlative analysis as a discussion point on the potential for using vector abundance as a proxy for hazard (see lines 724-34).

Line 316. “is an occurrence specific intercept”. Clarify what you mean by occurrence specific intercept since the term occurrence could be conflated with spatial location. Is this an observation level random effect for each month by location combination? I may have missed it but I don’t think the priors for this random effect are given later in the methods.

Response - This intercept was only added in the first tier of the model when modelling occurrence data. It is a single, fixed parameter that is only in the linear predictor for occurrence data. It acts as a varying intercept so that all occurrence data are modelled as a separate cluster to abundance data and therefore allows some flexibility in the joint modelling of both data types. An alternative interpretation is that it is an intercept parameter that is fixed at 0 for abundance data and allowed to vary for occurrence data. We have edited the text to read “is an occurrence data specific intercept”. We have added a couple of sentences to explain the method (see lines 389-93).

Line 369. Could temporal autocorrelation have been tested for shorter runs of data in simpler ways?

Response - For most studies, sampling over consecutive months did not occur (see S1 Table). However, we had performed temporal analysis of the data from the study with the longest consecutive duration (Samuel et al. 2016; study 16 in S1 Table) and found no evidence of temporal autocorrelation in the data when assessed using the autocorrelation function (ACF) to check for correlation between time lags. We did not include this information in the manuscript since it did not change our modelling approach.

Line 367. WAIC does not measure predictive ability but model parsimony. This sentence should be rephrased, including to inform the reader that lower WAIC = more parsimonious model.

Response - We have made the suggested edits.

Line 436: “The random selection of inferred absence data points was found to have no substantial (>2) impact on WAIC” This statement is not evidenced anywhere. The WAIC values are not comparable between these models since the underlying dataset is not the same. The best the authors can do is to show (in supplementary) that the delta WAIC values between the baseline, environmental, and seasonal models are still equivalently large.

Response - Thank you for your very useful comment. We have now provided this information in S3 Table and have referred to it in the text (see lines 528-30).

Line 461-463: Explain in the methods how you will interpret variable importance of the Bayesian Credible Intervals in the results.

Response - We have added some sentences in the method to explain the interpretation (see lines 467-71).

Line 465-466. “We found that the inclusion of a nonlinear effect for mean monthly temperature without a lag improved model predictive ability when compared to the nonlinear effect with two-month lagged temperature”. This statement is not evidenced by the results presented

Response - We have now provided a delta WAIC value to support this statement.

Lines 543-544 “assist in guiding interventions when long-term and large spatial scale surveillance data are not available or could not be practically acquired”

Response - We have now changed the focus of this sentence to focus on guiding surveillance efforts rather than interventions (see lines 642-6).

Links between rice cultivation and JE Line 586 “lead to changes in local ecology (e.g., biotic interactions such as competition and 587 predation)”.

Can you relate these findings to how resource use or dynamics of the key livestock and wildlife hosts for JE might respond to rice cycle dynamics? What types of empirical data would be needed to understand these relationships better?

Response - We have added some sentences expanding on these points (see lines 692-6).

Line 588 “predicted expansion of flooded areas for rice cultivation [89,90]”. Clarify whether this is a policy driven expansion, or whether you are referring to a climate impact prediction.

Response - We have explained predicted expansion is policy-driven to bolster food security.

Replace “my” with “we” where it appears.

Response - We have made these edits.

**References**

Lowe, Rachel, Sophie A. Lee, Kathleen M. O’Reilly, Oliver J. Brady, Leonardo Bastos, Gabriel Carrasco-Escobar, Rafael de Castro Catão, et al. 2021. “Combined Effects of Hydrometeorological Hazards and Urbanisation on Dengue Risk in Brazil: A Spatiotemporal Modelling Study.” The Lancet Planetary Health 5 (4): e209–19. <https://doi.org/10.1016/S2542-5196(20)30292-8>.

Lowe, Rachel, Bernard Cazelles, Richard Paul, and Xavier Rodó. 2016. “Quantifying the Added Value of Climate Information in a Spatio-Temporal Dengue Model.” Stochastic Environmental Research and Risk Assessment 30 (8): 2067–78. <https://doi.org/10.1007/s00477-015-1053-1>.

McElreath, Richard. 2020. Statistical Rethinking: A Bayesian Course with Examples in R and STAN. Second. Chapman and Hall/CRC.
