# Supplementary material for: Joint spatiotemporal modelling reveals seasonally dynamic patterns of Japanese encephalitis vector abundance across India
Source: PLoS Negl Trop Dis. 2022 Feb 22;16(2):e0010218. doi: 10.1371/journal.pntd.0010218 (PMC8896663; doi:10.1371/journal.pntd.0010218)
Supplement: Attachment — Submitted filename: ReviewComments.docx [file pntd.0010218.s012.docx]

I’ve read through the updated manuscript and the response to review comments. The manuscript now reads clearer, and the authors have fully explained, in their responses, answers to any queries I had. The study presents a good contribution to JE research, and I’d support publication in PLoS NTD without further need for review. I have detailed some minor comments below which the authors may wish to consider in a final draft of their manuscript.

**Intro**

93-96: this could be simplified to ‘…are important contributors to pathogen establishment (invasion?), transmission and persistence’.

111: ‘an…estimation**s**’

124: would just delete ‘large’ - ‘for national or regional scales’ – here and elsewhere would try and not use parentheses where possible to simplify text.

125: would delete ‘great’ here.

126: would argue here not ‘hazard’ but ‘abundance’ or components of hazard – as you make clear that it is difficult to estimate hazard

139: comma before however.

170: reference appears to be missing here.

**Methods**

215: suggest move to after line 202

235: suggest delete ‘To represent…at high altitudes’

277: why was two months chosen?

**Results**

516: would delete surprisingly.

578: can you say more about the parameter estimates of the best fitting model or present in a table? Even better would be to plot the data as a function of abundance and the fitted model. While the one-month lag abundance is better, it is hard for the reader to interpret how useful this is.

669: I think this should be S4 table.
