# Supplementary material for: Joint spatiotemporal modelling reveals seasonally dynamic patterns of Japanese encephalitis vector abundance across India
Source: PLoS Negl Trop Dis. 2022 Feb 22;16(2):e0010218. doi: 10.1371/journal.pntd.0010218 (PMC8896663; doi:10.1371/journal.pntd.0010218)
Supplement: Attachment — Submitted filename: Reviewer Responses_240122.docx [file pntd.0010218.s013.docx]

**Reviews of Manuscript No.:** PNTD-D-21-00888

**Title:** Joint spatiotemporal vector modelling reveals seasonally dynamic hazard patterns of Japanese encephalitis across India.

**Authors:** Lydia H.V. Franklinos, David W. Redding, Tim C.D. Lucas, Rory Gibb, Ibrahim Abubakar, Kate E. Jones.

We thank the editors and the two reviewers for taking the time to review our work again and for their positive feedback and helpful suggestions for improving our manuscript. Please find below our responses to the queries raised, shown in red, with reference to specific line numbers or figure/tables. All line numbers refer to the marked-up copy version of the revised manuscript.

**Reviewer #1:**

I’ve read through the updated manuscript and the response to review comments. The manuscript now reads clearer, and the authors have fully explained, in their responses, answers to any queries I had. The study presents a good contribution to JE research, and I’d support publication in PLoS NTD without further need for review. I have detailed some minor comments below which the authors may wish to consider in a final draft of their manuscript.

Response - Thank you for your review and suggestions which we believe have enhanced the manuscript.

**Intro**

93-96: this could be simplified to ‘…are important contributors to pathogen establishment (invasion?), transmission and persistence’.

Response - We have made the suggested edits.

111: ‘an…estimation**s**’

Response - We have made the suggested edits.

124: would just delete ‘large’ - ‘for national or regional scales’ – here and elsewhere would try and not use parentheses where possible to simplify text.

Response - We have made the suggested edits.

125: would delete ‘great’ here.

Response - We have made the suggested edits.

126: would argue here not ‘hazard’ but ‘abundance’ or components of hazard – as you make clear that it is difficult to estimate hazard

Response - We have made the suggested edits.

139: comma before however.

Response - We have made the suggested edits.

170: reference appears to be missing here.

Response – We have deleted the extra comma in the referencing (there was no missing reference).

**Methods**

215: suggest move to after line 202

Response - We believe the current order of the sentences clarifies the different processes of database creation and this would be confusing if the sentence in line 215 were moved to after line 202. Lines 201-210 outline the process of the literature search and lines 211-16 describes the extraction of data from the literature and the details of the final database.

235: suggest delete ‘To represent…at high altitudes’

Response - We have made the suggested edits.

277: why was two months chosen?

Response - We have explained that this lag was chosen to account for the period for mosquito larval habitat to increase and the development period of the mosquito (see lines 276-281).

**Results**

516: would delete surprisingly.

Response - We have made the suggested edits (see line 519).

578: can you say more about the parameter estimates of the best fitting model or present in a table? Even better would be to plot the data as a function of abundance and the fitted model. While the one-month lag abundance is better, it is hard for the reader to interpret how useful this is.

Response - We have provided a new figure (S5 Fig) as suggested plotting the fitted values against log vector abundance for the best-fitting model (model with one-month lag abundance). We have referred to this new figure in the results section (see lines 592-5).

669: I think this should be S4 table.

Response - We have made the suggested edits.

**Reviewer #2:**

The authors have addressed all of my original comments around the statistical methods. The statistical analysis is highly appropriate and very clearly articulated (e.g. random effects in the model, treatment of vector data, variable selection methods and accuracy/parsimony metrics, sensitivity analysis) and interpreted. The results and figures are clearly articulated.

The conclusions are well supported by the data analysis. I appreciated the authors refinement of their statements around the potential policy impact of their work and the additional discussion of the limitations of the analysis.

Response - Thank you for your review and comments which we believe have resulted in an improved manuscript.

I have some minor comments for the following sentences which the editors/authors can address or not:

Vector abundance i.e., the number of individuals in a site at a given time, and seasonality i.e., intra-annual change in abundance, are important contributors to many epidemiological factors that influence MBD hazard; these factors include pathogen establishment, persistence and transmission [6,8,9].

Please rewrite to clarify the meaning here on how you are categorising the factors affecting MBD hazard. For example, it is strange to have two i.e. in one sentence and the sub-clause “important contributors to many epidemiological factors that influence hazard” is unclear.

Response – We have simplified this sentence as suggested by Reviewer 1 so that we clarify that vector abundance and seasonality are important to pathogen establishment, persistence and transmission (see lines 93-96).

Line 99. Arguably longer vector seasons could also increase contact rates between vectors and hosts, by increasing the duration of seasonal overlap.

Response – We had edited lines 99-101 to include this point.

Line 309 “exposure to JEV however, other factors”. Suggest splitting into two sentences by inserting full stop after JEV here.

Response – We have rewritten the sentence to improve the flow (see lines 139-142).

Line 640 “Surprisingly, we found that the annual area under rice cultivation was negatively associated with vector abundance”. Could clarify that this was not significant.

Response – We found that the annual area under rice cultivation had a significant negative effect on vector abundance although it had wide CIs (see Figure 2B). We think that perhaps you are referring to the proportion of agricultural land was found to be non-significant. We have now referred to the wide CIs associated with the annual area under rice cultivation result (see line 648).

The authors have made substantial efforts to address all the comments and the paper will make an excellent contribution the field.

Response – Thank you for noting the contribution of our work to the field.
